# Knowledge, practice and associated factors of nurses towards prevention of catheter-associated urinary tract infection in intensive care unit of public hospitals administered by Federal Government in Addis Ababa, Ethiopia: a cross-sectional institutional-based study

**DOI:** 10.1186/s12912-022-00968-1

**Published:** 2022-07-15

**Authors:** Tilahun Teshager, Heyria Hussien, Merahi Kefyalew, Fenta Wondimneh, Indeshaw Ketema, Sisay Habte

**Affiliations:** 1 School of Nursing and Midwifery, College of Health and Medical Sciences, Haramaya University, Harar, Ethiopia; 2 Department of Emergency Medicine, School of Medicine, College of Health Sciences, Addis Ababa University, Addis Ababa, Ethiopia

**Keywords:** Catheter-associated urinary tract infection, Knowledge, Practice, Nurses

## Abstract

**Background:**

Urinary catheterization is one of the most common procedures performed in hospitals specifically, in the intensive care units and is associated with a high risk for acquired urinary tract infections. More than 70% of acquired urinary tract infections are due to catheter use. Nurses are the primary healthcare providers responsible for inserting and maintaining urinary catheters. The data regarding nurses’ knowledge, practice and associated factors towards prevention of catheter-associated urinary tract infections are limited in Ethiopia. Therefore, this study aimed to assess the knowledge, practice, and associated factors of nurses towards prevention of catheter-associated UTIs in the Intensive Care Unit (ICU) of public hospitals in Addis Ababa, Ethiopia.

**Methods:**

An institutional-based descriptive cross-sectional study was conducted from March 01 to April 15, 2021, among nurses working in the ICU of public hospitals in Addis Ababa, Ethiopia. All 204 nurses working in the ICU of four public hospitals were included in the study using the census sampling method. Data were collected using a pretested self-administered semi-structured questionnaire. Data were cleaned and entered into Epi data version 4.6 and analyzed using Statistical Package for Social Sciences version 26.0. Pearson Chi-square and Fischer exact tests were performed to see the association between independent and dependent variables. The level of significance is considered at *P*-value less than 0.05.

**Results:**

A total of 184 nurses participated in the study, making a response rate of 90.2%. The mean (±SD) age of the study participant was 29.07(±4.78). The study findings showed that more than half (63.04%) of nurses had poor knowledge and 88(47.83%) of nurses had poor practice towards prevention of catheter-associated UTIs. In this study, there was a statistically significant association between professional work experience and nurses’ knowledge in preventing catheter-associated UTIs (at *P*-value = 0.031).

**Conclusion:**

In this study, nurses’ knowledge and practice towards the prevention of catheter-associated urinary tract infection was relatively poor. Professional work experience had a significant statistical association with the level of knowledge. Therefore, increasing the knowledge of nurses through appropriate educational programs and training on the preventive measures of device-associated infections was recommended to prevent catheter-associated UTIs.

## Introduction

Catheter-associated Urinary Tract Infections (CAUTIs) is a urinary tract infections (UTIs) in a patient with a catheter present or within 48 h of catheter removal [1]. It affects any part of the urinary system, including the kidney, ureter, bladder, and urethra [2–4]. CAUTIs are acquired when the insertion of a urinary catheter unnecessarily and the presence of a catheter for a long period in the bladder [5, 6]. More than 70% of UTIs are acquired due to the use of indwelling urinary catheters and unnecessary instrumentation [7].

CAUTIs are caused by organisms such as E.coli(21.4%), Enterococcus(14.9%), *Pseudomonas Aeruginosa*(10%), Klebsiella Pneumonia(7.75%) and Enterobacter (4.15%) [8, 9]. The risk factors associated with acquiring CAUTIs were; being female, prolonged duration of catheterization, immune-compromised patients, advanced age, and prolonged ICU stay [10].

CAUTIs can lead to different complications such as; prostatitis, epididymitis, bladder spasm, orchitis in males and cystitis, pyelonephritis, urosepsis, endocarditis, endophthalmitis, meningitis, and bloodstream infections [11]. It has been associated with increased morbidity, mortality, high financial cost, and prolonged length of stay in the hospital [11–13]. According to the CDC guideline, catheterized patients have more than 80% of developing urinary tract infections when compared to non-catheterized patients, with a higher incidence in developing countries compared to developed countries [14, 15].

The knowledge and practice of nurses towards prevention of CAUTIs was poor in developing countries. For instance, in India (58.8%) [9], Philippines (70%) [16], Rwanda (64.52%) [15] and Egypt (83.94%) [17]. The guidelines for the prevention of CAUTIs have recommended proper catheter use, aseptic insertion, good maintenance, and cultural and behavioral change in the health care units are vital in avoiding catheter-associated UTIs [18, 19].

Shortening the duration of catheterization, avoiding unnecessary catheterization, preparing and implementing standard infections control protocols and using sterile precautions in the insertion and maintenance of indwelling urinary catheters can reduce catheter-related complications [12, 20].

Nurses are the primary healthcare providers responsible for inserting and maintaining urinary catheters, as well as the production of desired outcomes by which they follow the available guidelines, protocols, and standards during catheter insertion and catheter cares [21]. The evidence indicates that factors such as the availability of resources and presence of guidelines in the health facility have a positive impact on the knowledge and practice of nurses towards prevention of CAUTIs [22].

In the developing countries, the incidence of CAUTIs approximately ranged from 10 to 35 per 1000 patients [10]. Similarly, in African countries, including Ethiopia, CAUTIs are the most common healthcare-associated infections accounting for 80% of hospital-acquired infections [15]. Despite the high incidence of CAUTIs in Ethiopia, the data regarding knowledge, practice and associated factors of nurses towards prevention of CAUTIs are limited. Therefore, this study aimed to assess the knowledge, practice and associated factors of nurses towards prevention of CAUTIs in the Intensive Care Unit at public hospitals in Addis Ababa, Ethiopia**.**

## Methods and materials

### Study settings and period

The study was conducted at four (4) public hospitals administered by the federal government in Addis Ababa, Ethiopia from March 01 to April 15, 2021. Addis Ababa is the capital city of Ethiopia which is located in the central part of Ethiopia. There are five (5) public hospitals administered by the federal government in Addis Ababa, namely Tikur Anbessa Specialized Hospital (TASH), Alert Trauma center Hospital, St. Paulo’s Specialized Hospital, St. Petros Referral Hospital and Amanuel Referral Hospital.

Tikur Anbessa Specialized Hospital has 52 nurses working in the ICU, Alert Specialized Hospital has 27 nurses working in the ICU, St. Paulo’s Specialized Hospital has 88 nurses working in the ICU, and St. Petros Referral Hospital has 37 nurses working in the ICU currently. Amanuel referral Hospital has no Intensive care unit. Hence, a total number of nurses working in the ICU of public hospitals administered by the federal government were 204.

### Study design and population

An institutional-based cross-sectional study was conducted from March 01 to April 15, 2021, among nurses working in the ICU of public hospitals administered by the federal government in Addis Ababa, Ethiopia. All nurses working in the ICU of each public hospital who were available during the data collection period were included in the study. Nurses who were on maternal leave, sick leave, and severely sick were excluded.

### Sample size and sampling procedure

All nurses working in the ICU of four public hospitals administered by the federal government in Addis Ababa and fulfilled inclusion criteria were included in the study using the census sampling technique.

### Measurements

#### Nurses’ knowledge

Measured by 8 knowledge related questions with multiple choices that have four responses. Nurses who chosen a correct answer was given a score of ‘1’ point, and ‘0’ point if not. A total score ranged from 0 to 8 . Nurses who scored 71% and above the score of knowledge related questions were categorized as having “good knowledge”, whereas those who scored below 71% were categorized as having “poor knowledge” [4].

#### Nurses’ practice

Measured by 18 practice related questions with ‘yes/no’ responses. Nurses who chosen a correct answer was given a score of ‘1’ point, and ‘0’ point if not. A total score ranged from 0 to 18. Nurses who scored 79.9% and above the score of practice related questions were categorized as “good practiced”, whereas those who reported below 79.9% were categorized as “poor practiced” [15].

### Data collection tool and techniques

Data were collected using a pretested self-administrated semi-structured questionnaire which is adapted from different literature [15, 17]. The questionnaire was prepared in English language and consisted of nurses’ socio-demographic characteristics (age, gender, educational level, and years of experience), knowledge and practice towards prevention of CAUTIs and institutional factors related questions. The questionnaire was validated by following the face validity method. To test the reliability of the tool, Cronbach alpha was calculated and the value was 0.77 which is in an acceptable range.

### Data quality management

A pretested self-administered semi-structured data collection tool was adopted to ensure data quality. One day training was given for data collectors and supervisors on the objectives of the study and other related issues. A pretest was done on 5% of the sample size before the actual data collection period to check for reliability and validity of data collection tools. The questionnaires were reviewed and checked for completeness, accuracy and consistency by data collectors and supervisors on daily basis.

### Data processing and analysis

The collected data were coded, categorized and entered into Epi-data Version 4.6.0 and analyzed by using Statistical Package for Social Science (SPSS) version 26.0. Descriptive findings were expressed as frequency percentages, mean, and standard deviation. Chi-square (X^2^) and Fischer exact tests were used to test for the significance of association between the independent and outcome variables. The level of significance was considered at a *P*-value < 0.05.

## Results

### Socio-demographic characteristics of nurses

Out of 204 study participants, a total of 184 nurses participated in the study, making a response rate of 90.2%. Among the study participants, more than half (53.3%) were female. The mean (+SD) age of the study participants was 29.07(±4.78). As depicted in Table 1 below, the majority, 113(61.4%) of the study participants were in age group of 20–29 years. Nearly three-fourths (74.5%) of the study participants had Bachelor of Science degrees. Half (50%) of the study participants had 1–5 years’ work experience. More than half (51.6%) of the study participants were married. Nearly one-third (35.9%) of the study participants were working in the pediatric ICU.

**Table 1:** Socio-demographic characteristics of nurses working in the ICU of public hospitals administered by the federal government in Addis Ababa, Ethiopia, 2021 (*N* = 184)

Variables	Frequency (percent)
**Sex of participants**	
Male	86(46.7%)
Female	98(53.3%)
**Age (**mean **= 29.07** and SD **= ± 4.78**)	
20–29 years	113(61.4%)
30–39 years	61(33.2%)
> 40 years	10(5.4%)
**Level of education/Qualification**	
Diploma	3(1.6%)
Bachelor of Science degree	137(74.5%)
Master of Science degree	44(23.9%)
**Professional experience**	
**<** 1 year	14(7.6%)
1 to 5 years	92(50%)
6 to 10 years	53(28.8%)
> 10 years	25(13.6%)
**Marital status**	
Single	83(45.1%)
Married	95(51.6%)
Divorced	6(3.3%)
**Current working department**	
Adult medical ICU	57(31.0%)
Adult surgical ICU	61(33.2%)
Pediatrics ICU	66(35.8%)

### Knowledge of nurses towards prevention of CAUTIs

**Fig. 1:**
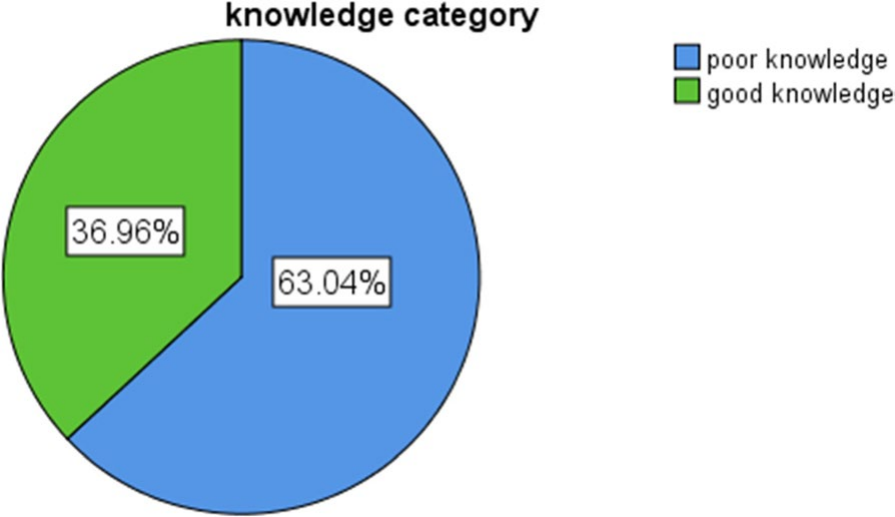
Nurses’ knowledge towards prevention of CAUTIs among nurses working in the ICU at public hospitals administered by the federal government in Addis Ababa, Ethiopia, 2021 (*N* = 184)

In this study finding, as shown in Fig. 1 below, nearly two-thirds (63.04%) of the study participants had poor level of knowledge, while 68(36.96%) had good level of knowledge towards prevention of CAUTIs. The mean knowledge score of the study participant was 5.16(SD ± 1.25). As depicted in Table 2 below, the majority, 123(71.7%) correctly answered urinary catheter insertion requires an aseptic technique with sterile equipment. Similarly, nearly two-thirds (63%) knew an appropriate indication for indwelling urinary catheterization. Two-thirds (66.8%) knew CDC guidelines for the prevention of CAUTI which advise that the catheter should be removed within 24 h in postoperative patients. More than half (53.8%) correctly identified changing the urinary catheters or drainage bags must be performed at routine and fixed intervals, while 107(58.2%) identified prolonged time of catheterization as a risk factor for CAUTI. More than two-thirds (67.4%) knew elderly patients above 65 years and women were at high risk for CAUTIs and the majority, 129(70.1%) knew that hypertension was not a complication of CAUTIs.

**Table 2:** Nurses’ self-reported level of knowledge on each item of knowledge questions in the ICU of public hospitals administered by the federal government in Addis Ababa, Ethiopia, 2021 (*N* = 184)

Items	Yes	No
	N (%)	N (%)
**Which is not an indication for indwelling urinary catheterization?**		
Inserting catheter for identifying the color of urine	116 (63%)	68 (37%)
**Which one is the proper method of indwelling urinary catheterization?**		
Using aseptic technique with sterile equipment	132(71.7%)	52(28.35)
**In postoperative patients, a catheter is advised to be removed preferably within,**		
24 h	123(66.8%)	61(33.2%)
**If an indwelling urinary catheter is obstructed, what will be the next steps?**		
Change the catheter immediately	121(65.8%)	63(34.2%)
**One is not a nursing achievement to prevent CAUTIs?**		
Changing the urinary catheters or drainage bags only at routine and fixed intervals	99(53.8%)	85(46.2%)
**Which one among the following is a risk factor for CAUTI?**		
Prolonged time of catheterization	107(58.2%)	77(41.8%)
**Which patient is at high risk for developing CAUTI among the following?**		
Elderly patients above 65 years and women	124(67.4%)	60(32.6%)
**All the following are complications of CAUTI except,**		
Hypertension	129(70.1%)	55(29.9%)

### Practice of nurses towards prevention of CAUTIs

**Fig. 2:**
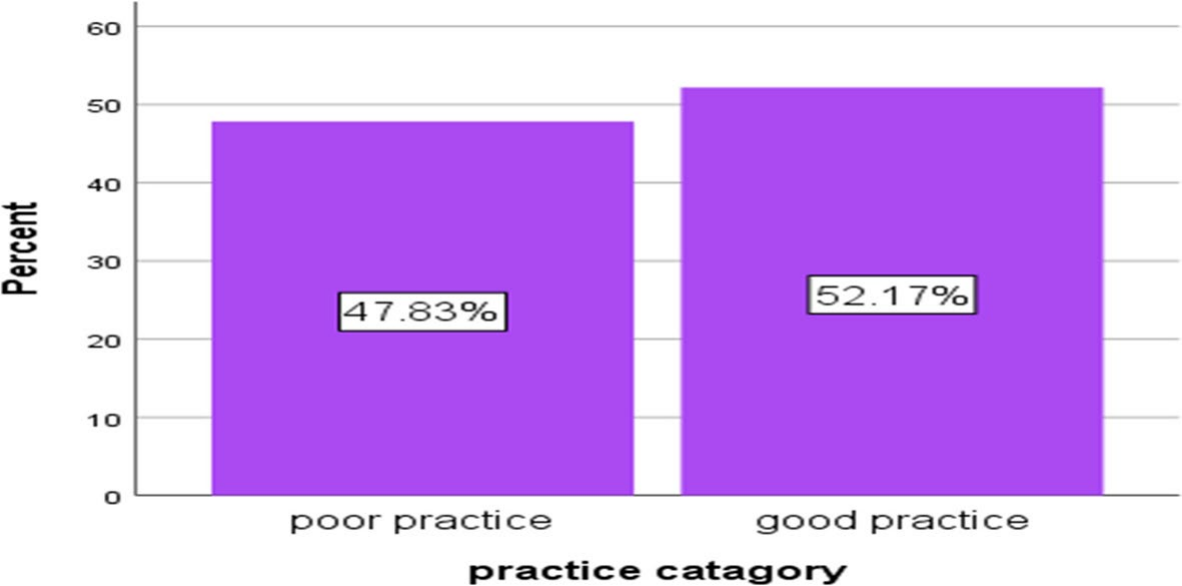
Nurses’ levels of practice towards prevention of CAUTIs in the ICU of public hospitals administered by the federal government in Addis Ababa, Ethiopia, 2021 (*N* = 184)

As depicted in Fig. 2 below, more than half (52.17%) of the respondents had good level of practice, while 88 (47.83%) had poor level of practice for the prevention of CAUTIs. The mean practice score of the study participant was 13.86(SD ± 3.59). As shown in Table 3 below, among the study participants, two-thirds (63%) practiced hand washing before catheter insertion, while greater than three-fourths (78.8%) practiced hand washing before and after catheter insertion. The majority, 155(84.2%) used an aseptic technique, and 150(81.5%) used sterile equipment like sterile gloves, drapes, sponges, and solution while performing catheterization. The majority, 158(85.9%) always kept the collecting bag below the bladder, 152(82.6%) kept the catheter and collecting tube free from kinking, and 130(70.7%) maintained the closed system all the time while doing routine catheter care.

**Table 3:** Nurses’ self-reported level of practice on each item of practice questions in the ICU of public hospitals administered by the federal government in Addis Ababa, Ethiopia, 2021 (*N* = 184)

Items	Yes	No
	N (%)	N (%)
**When do you perform hand hygiene?**		
Hand washing before and after catheter insertion	145 (78.8%)	39 (21.2%)
Hand washing before catheter manipulation	116 (63%)	68 (37%)
Hand washing after catheter insertion	150 (81.5%)	34(18.5%)
**Do you use the following while doing catheterization?**		
Use sterile equipment like sterile gloves, drape, sponges	168 (91.3%)	16 (8.7%)
Use an aseptic technique to insert the catheter	155 (84.2%)	29(15.8%)
Clean the urethra meatus with an antiseptic solution	135 (73.4%)	49 (26.6%)
Use a single packed lubricant jelly	126(68.5%)	58(31.5%)
Secure indwelling catheter properly after insertion to prevent movement and urethral traction	139(75.5%)	45(24.5%)
Being familiar with the use of catheter alternatives like condom or adsorbent pads	135 (73.4%)	49 (26.6%)
**Do you do the following while routine catheter cares?**		
Keep the catheter and collecting tube free from kinking	152 (82.6%)	32 (17.4%)
Always keep the collecting bag below the bladder	158 (85.9%)	26 (14.1%)
Empty collecting bag regularly & use a separate collecting jug for each patient	131 (71.2%)	53 (28.8%)
Maintain the closed system all the time	130 (70.7%)	54 (29.3%)
Use PPE as appropriate, during manipulation of the catheter	153 (83.2%)	31 (16.8%)
**Do you use the appropriate techniques for catheter removal?**		
Protect yourself during catheter removal	161 (87.5%)	23 (12.5%)
Use a syringe on the catheter to deflate the balloon	173 (94%)	11 (6%)
Use a release technique for any catheter fixation for easy removal	127 (69%)	57 (31%)
Removed date recorded	98 (53.3%)	86 (46.7%)

### Institutional factors of nurses towards prevention of CAUTIs

According to the reports of this study, as shown in Table 4 below, more than half (51.6%) of the study participants reported that guidelines/protocols were available in their working units. Seventy-one (38.6%) of the study participants have got on job training towards prevention of CAUTIs. More than half (55.4%) of the study participants had hand washing facility in their working area, whereas 100(54.3%) of the study participants reported they had enough supplies at their institution.

**Table 4:** Institutional factors of nurses in the prevention of CAUTIs in the ICU of public hospitals administered by the federal government in Addis Ababa, Ethiopia, 2021 (*N* = 184)

Variables	Frequency (%)	
	Yes	No
Do you have guidelines for the proper insertion of a foley catheter?	95(51.6%)	89(48.4%)
Have you ever got on job training about catheterization?	71(38.6%)	113 (61.4%)
Do you have enough supply for catheterization at your institution?	100(54.3%)	84 (45.7%)
Do you have a handwashing facility in your work area?	102(55.4%)	82 (44,6%)

### Factors associated with nurses’ knowledge and practice towards prevention of CAUTIs

The Pearson chi-square and Fischer exact tests were used to determine the association between independent and outcome variables. As depicted in Table 5 below, professional work experiences (*P*-value = 0.031) had a statistically significant association with nurses' knowledge towards prevention of catheter-associated UTIs. As shown in Table 6 below, there was no statistical association between professional work experiences and nurses' practice in the prevention of CAUTIs (*P*-value = 0.700). Socio-demographic characteristics (age, level of education, current working department) and institutional factors (training, guidelines, and hand washing facility) had no statistical association with nurses’ knowledge and practice towards prevention of CAUTIs.

**Table 5:** Factors associated with nurses' knowledge towards prevention of CAUTIs in the ICU of public hospitals administered by the federal government in Addis Ababa, Ethiopia, 2021. (*N* = 184)

	Levels of Knowledge					
	Good knowledge		Poor Knowledge		Test used	***P***-value
**Sex of respondents**	**N**	**%**	**N**	**%**	**Pearson chi-square test**	0.542
Male	34	39.5	52	60.5		
Female	34	34.7	64	65.3		
**Age category**					**Fischer**	0.163
20–29 years	37	32.7	76	67.3	**exact test**	
30–39 years	25	41.0	36	59.0		
40–49 years	6	60.0	4	40.0		
**Level of education**					**Fischer**	0.204
Diploma	0	0.0	3	100.0	**exact test**	
BSc	48	35.0	89	65.0		
MSc	20	45.5	24	54.5		
**Professional experience**					**Fischer**	**0.031**
Less than 1 year	3	21.4	11	78.6	**exact test**	
1–5 years	27	29.3	65	70.7		
6–10 years	24	45.3	29	54.7		
11–15 years	8	50.0	8	50.0		
16–20 years	3	50.0	3	50.0		
Above 21 years	3	100.0	0	0.0		
**Marital status**					**Fischer**	0.186
Single	26	31.3	57	68.7	**exact test**	
Married	41	43.2	54	56.8		
Divorced	1	16.7	5	83.3		
**Current working dept.**					**Pearson chi-**	0.317
Adult medical ICU	18	31.6	39	68.4	**square test**	
Adult surgical ICU	21	34.4	40	65.6		
Pediatric ICU	29	43.9	37	56.1		
Your facility has guidelines/ protocols for proper insertion of a foley catheter					**Pearson chi-square test**	1.000
**Yes**	35	36.8	60	63.2		
**No**	33	37.1	56	62.9		
Have you ever got on job training about catheterization?					**Pearson chi-square test**	0.211
**Yes**	22	31.0	49	69.0		
**No**	46	40.7	67	59.3		
Do you have enough supply for catheterization at your institution (like a lubricant, sterile drapes...)?					**Pearson chi-square test**	0.878
**Yes**	36	36.0	64	64.0		
**No**	32	38.1	52	61.9		
Do you have a hand washing facility (soap, water, Antiseptic) in your work area?					**Pearson chi-square test**	0.284
**Yes**	34	33.3	68	66.7		
**No**	34	41.5	48	58.5		

*P*-value is significant at *p* < 0.05

**Table 6:** Factors associated with nurses' level of practice towards prevention of CAUTIs in the ICU of public hospitals administered by the federal government in Addis Ababa, Ethiopia, 2021. (*N* = 184)

Variables	Level of practice					
	Good practice		Poor practice		Test used	***P***-value
**Sex of respondents**	**N**	**%**	**N**	**%**	**Pearson chi-**	0.239
Male	49	57.0	37	43.0	**square test**	
Female	47	48.0	51	52.0		
**Age category**					**Fischer exact**	0.680
20–29 years	56	49.6	57	50.4	**test**	
30–39 years	34	55.7	27	44.3		
40–49 years	4	40.0	6	60.0		
**Level of education**					**Fischer exact**	0.505
Diploma	2	66.7	1	33.3	**test**	
BSc degree	68	49.6	69	50.4		
MSc degree	26	59.1	18	40.9		
**Professional experience**					**Fischer exact**	0.700
Less than 1 year	6	42.9	8	57.1	**test**	
1–5 years	47	51.1	45	48.9		
6–10 years	29	54.7	24	45.3		
11–15 years	8	50.0	8	50.0		
16–20 years	3	50.0	3	50.0		
Above 21 years	3	100.0	0	0.0		
**Marital status**					**Fischer exact**	0.337
Single	43	51.8	40	48.2	**test**	
Married	48	50.5	47	49.5		
Divorced	5	83.3	1	16.7		
**Current working dept.**					**Pearson chi-**	0.807
Adult medical ICU	29	50.9	28	49.1	**square test**	
Adult surgical ICU	34	55.7	27	44.3		
Pediatrics ICU	33	50.0	33	50.0		
Your facility has guidelines/ protocols for proper insertion of Foley catheter					**Pearson chi-square test**	0.076
**Yes**	56	68.9	39	41.1		
**No**	40	44.9	49	55.1		
Have you ever got on job training about catheterization?					**Pearson chi-square test**	0.449
**Yes**	40	56.3	31	43.7		
**No**	56	49.6	57	50.4		
Do you have enough supply for catheterization at your institution (like lubricant, sterile drapes)?					**Pearson chi-square test**	0.100
**Yes**	52	52.0	48	48.0		
**No**	44	52.4	40	47.6		
Do you have a hand washing facility (soap, water, antiseptic) in your work area?					**Pearson chi-square test**	0.300
**Yes**	57	55.9	45	44.1		
**No**	39	47.6	43	52.4		

*P*-value is significant at *p* < 0.05

### Correlation between nurses’ knowledge and practice towards prevention of CAUTIs

Regarding the association between nurses’ level of knowledge and practices towards CAUTIs prevention, as depicted in Table 7 below, it was observed that there was no statistical association between nurses’ knowledge and practice towards CAUTIs prevention with a *p*-value = 0.450.

**Table 7:** Correlation between nurses’ knowledge and practice towards Prevention of CAUTIs in the ICU of public hospitals administered by the federal government in Addis Ababa, Ethiopia, 2021. (*N* = 184)

	Level of nurse’s knowledge and Practice correlation	
		Level of Practice
**Level of Knowledge**	Spearman correlation	0.057
	*P*-value	0.450

## Discussion

Hospital-acquired infections (HAIs) constitute major public health problems worldwide; among which Catheter-Associated Urinary Tract Infections (CAUTIs) are the most common [23]. More than 70% of UTIs are reported because of indwelling catheters and unnecessary instrumentation [7, 13]. Nurses are the primary healthcare providers responsible for inserting and maintaining urinary catheters, as well as the production of desired outcomes by which they follow the available guidelines, protocols, and standards during catheter insertion and catheter cares [15, 21]. In recent years, many efforts have done to change the behaviors of nurses towards the prevention of CAUTIs and limit the unnecessary use of IUC [3]. Therefore, this study aimed to assess the knowledge, practice and associated factors towards prevention of CAUTIs among nurses working in the intensive care units at public hospitals in Addis Ababa, Ethiopia.

In this study finding, only 36.96% of nurses had good knowledge towards prevention of Catheter-associated UTIs. This finding is in line with the study conducted in India [9], Egypt [17] and Rwanda [15]. The possible reason might be due to the same study design used (cross-sectional) and study subjects (nurses). In contrast, this finding is relatively higher than the study conducted in Philippines at Iloilo city [16]. This might be due to the difference in the sample size, the previous study done in Philippines had a lower sample size (*n* = 30). But, the sample size in the current study was 204, which is higher than that of the previous study. However, this finding is lower than the findings of the studies conducted in Pakistan at public hospitals in Lahore [24] and Iran [25]. This discrepancy might be due to the differences in the health facility setup and availability of the guidelines.

The current study revealed that more than half (52.2%) of the study participants had good practice towards prevention of Catheter-associated UTIs. This finding is higher than the studies conducted in Pakistan [26], Philippines [16] and Egypt [17]. This discrepancy might be due to the educational status of the study participants, the difference in the study setting and the sample size. In the previous studies, Pakistan (73.4%), Philippines (66.7%) and Egypt (62.1%) were diploma nurses. But, in the current study, most nurses were a Bachelor of Science degree (79.5%), and the previous study had a lower sample size; Pakistan (184), Philippines (30), and Egypt (137). But, in the current study, the sample size was 204. However, this finding is lower than a study conducted in Rwanda [15]. This discrepancy might be due to the differences in the tool used to rate the outcome, availability of infection prevention protocols and guidelines and availability of supplies in their working units.

In the current study, nearly half (51.6%) of the study participants reported that guidelines/protocols were available in their working units. This finding is consistent with the study conducted in Pakistan at public hospitals in Lahore [24]. The possible reason for the consistency might be due to the same study setting since both studies were conducted in ICU, where critical patients were treated, and the study subjects (nurses). In this study finding, more than one-third (38.6%) of nurses had got on job training on how to prevent infections during catheterization. This finding is inconsistent with the findings of the studies conducted in Egypt [17] and Rwanda [15]. This discrepancy might be due to the difference in the institutional facility and resource limitation, in a country like Ethiopia with low socio-economic status, there is resource limitation in the health facility. However, more than half of the study participants reported that there were enough supplies (like a lubricant, solution for cleansing, sterile drapes), and hand washing facility in their working area. This study is consistent with the study conducted in Pakistan at public hospitals in Lahore [24]. The possible reason for the consistency might be due to the same study setting and study subjects (nurses).

According to the reports of this study, nurses’ level of knowledge is not statistically associated with nurses’ level of practice (*P*-value = 0.450). This finding agrees with the study conducted in Pakistan at Iloilo city [16] and Egypt [17]. The possible justification for this similarity might be due to similar study design used and poor nurses’ level of knowledge and practice in both studies. However, in the current study, there was a statistically significant association between professional work experience and nurses’ level of knowledge (at *P*-value = 0.031). This finding is in line with the study conducted in Pakistan [27]. The possible justification for the similarity might be due to the fact that work experience increases nurses’ level of knowledge towards prevention of CAUTIs. This implies that nurses can learn or gain knowledge from their experience if they stayed in the unit for a prolonged time. Thus, this study concluded that there is no impact of socio-demographic characteristics (except professional experience) and institutional factors on nurses’ knowledge and practice towards prevention of Catheter-associated UTIs.

## Limitations

The study had some limitations. Primarily, a cross-sectional study was used and it did not show a causal relationship between study variables. The study could not observe the actual nurses’ practices towards prevention of CAUTIs. Finally, the questionnaire was prone to social desirability bias; because everyone does not want to expose once inability to practice.

## Conclusion

In this study, nurses’ knowledge and practice towards prevention of catheter-associated UTIs was relatively poor. Nurses’ professional work experience had a significant statistical association with the level of knowledge towards prevention of CAUTI. Therefore, we recommended increasing the knowledge of nursing staff through appropriate educational programs and training regarding the preventive measures of device-associated infections on how to avoid unnecessary urinary catheter use. We also recommended further studies with large sample sizes need to investigate factors affecting nurses’ knowledge and practice towards prevention of Catheter-associated UTIs.

## Data Availability

All the data supporting the study findings are within the manuscript. Additional detailed information and raw data are available from the corresponding author on reasonable request.

## Abbreviations

AOR: Adjusted Odds Ratio
CAUTIs: Catheter-associated urinary tract infections
CDC: Centers for disease control and prevention
CI: Confidence Interval
HAI/HCAI: Healthcare-associated infection
ICU: Intensive care unit
IUC: Indwelling urinary catheter
SPSS: Statistical Package for Social Sciences
UTI: Urinary tract infection
